# Use of contemporary technologies and new materials in undergraduate Endodontics teaching

**DOI:** 10.4317/jced.57795

**Published:** 2021-04-01

**Authors:** Mari-Carmen Jiménez-Sánchez, Juan J. Segura-Egea, Alicia Zarza-Rebollo, Victoria Areal-Quecuty, Paloma Montero-Miralles, Jenifer Martín-González, Daniel Cabanillas-Balsera

**Affiliations:** 1 DDS, MSc, PhD, Materials Science Institute of Sevilla (ICMS), Joint CSIC-University of Sevilla Center, 41092 Sevilla, Spain; 2 MD, DDS, PhD, Professor, Department of Stomatology, Section of Endodontics, School of Dentistry, University of Sevilla, C/ Avicena s/n, 41009-Sevilla, Spain; 3 DDS, MSc, Doctoral fellow, Department of Stomatology, Section of Endodontics, School of Dentistry, University of Sevilla, C/ Avicena s/n, 41009-Sevilla, Spain; 4 DDS, MSc, Doctoral fellow, Department of Stomatology, Section of Endodontics, School of Dentistry, University of Sevilla, C/ Avicena s/n, 41009-Sevilla, Spain; 5 DDS, MSc, PhD, Professor of the Master in Clinical Endodontics, University of Sevilla, C/ Avicena s/n, 41009-Sevilla, Spain; 6 DDS, PhD Associate Professor, Department of Stomatology, Section of Endodontics, University of Sevilla, C/ Avicena s/n, 41009-Sevilla, Spain; 7 DDS, MSc, Doctoral fellow, Department of Stomatology, Section of Endodontics, School of Dentistry, University of Sevilla, C/ Avicena s/n, 41009-Sevilla, Spain

## Abstract

**Background:**

This study aims to analyze the use of contemporary technologies and materials in undergraduate endodontic teaching in Spain.

**Material and Methods:**

The survey was sent to the undergraduate endodontic programme leads in the 23 Spanish dental schools. The survey asked about the use of magnification, ultrasonic devices, electronic apex locator, rotary instruments, root-filling techniques, and bioceramic cements in the teaching of endodontics.

**Results:**

The response rate was 91%, and the final number of schools included in the study was twenty. Only two schools (10%) used magnification (loupes or operative microscope). Five schools (25%) used ultrasonic devices to prepare the access cavity, and four (20%) to activate the irrigation solution. In 14 dental schools (70%) no type of ultrasonic instrument was used. Electronic apex locators in working length determination was used in 19 schools (95%). All schools used rotary instrumentation in the teaching of endodontics, and 45% of schools used reciprocating instruments. Five schools (25%) used warm vertical compaction technique, four (20%) single cone gutta-percha technique, and four (20%) thermoplastic injection techniques. No school used carrier-based gutta-percha. Bioceramic cements were used in 19 of the schools (95%).

**Conclusions:**

Spanish dental schools have incorporated some of the new endodontic technologies and materials, including the electronic apex locator, rotary instruments, and the new bioceramic cements; however, the modern root filling techniques, magnification, and ultrasonic instruments are not yet used in most dental schools.

** Key words:**Bioceramic materials, dental schools, endodontic curriculum, magnification, online survey, rotary instrumentation, ultrasonic devices.

## Introduction

Graduate in dental schools must acquire the knowledge and skills necessary to perform endodontic procedures with an adequate level of competence (1). Technical standards of endodontic treatment are associated with quality and quantity of undergraduate education in Endodontology (2,3). For this reason, it is very important that new technologies and new materials are incorporated into undergraduate teaching of endodontics. Only in this way will it be guaranteed that the graduate has the necessary skills to be able to perform endodontic treatments complying with the required standards.

The European Society of Endodontology (1) and the Association for Dental Education (4,5) recommended the use of contemporary technologies and new materials in endodontic undergraduate teaching. The incorporation into endodontic clinical practice of electronic apex locators, ultrasound, rotary instruments, magnification and continuous heat wave obturation techniques, and new bioceramic materials, has led to a substantial improvement in endodontic technique, better treatment outcomes, and greater comfort for the endodontist and patients.

In Spain, the endodontic teaching in each one of the 23 existing dental schools, 12 in public universities and 11 in private universities, is carried out according to the curriculum approved by the University and supervised by the National Agency for Quality Assessment and Accreditation (ANECA).

However, there is no data on actual use of contemporary techniques and new endodontic materials in Spanish dental schools. The objective of this study was to analyze by means of a survey, the current use of contemporary technologies and new materials in the undergraduate endodontic teaching in Spain.

## Material and Methods

An online survey was used, including multiple-choice questions, with more than one answer selected when appropriate. The topics included in the questions were the following (Table 1): 1) Use of magnification, 2) Use of ultrasonic instruments, 3) Use of electronic apex locators, 4) Use of rotary systems in root canal instrumentation, 5) Use of filling techniques, other than lateral compaction, for root canal filling, and 6) Use of advanced endodontic materials such as MTA or Biodentine.

**Table 1:**
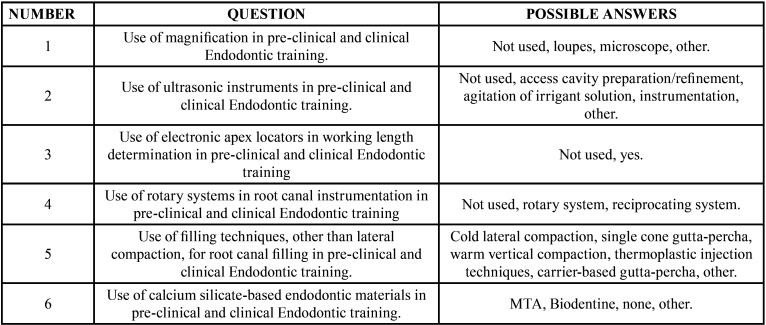
Topics investigated in the survey on the use of modern technologies in undergraduate Endodontic training in Spain (and possible answers).

The contact details of the professors in charge of teaching endodontics in each dental school were obtained online, from school web pages, and through personal contacts. All the undergraduate endodontic program leaders were sent an email explaining the objective of the study, together with a link to answer the survey through Google. The survey opened on December 23, 2019, the same day the first email with the link was sent. A reminder email was sent 1 week after initial contact. The data was obtained directly from Google, which generated it maintaining anonymity. Using the Excel program, simple descriptive statistics were generated for each item in the survey.

## Results

Of the 23 dental schools that were asked to collaborate in the study, 21 responded to the survey (91.3%). Given that one of the schools was excluded because it had not completed its dental degree studies and was not yet teaching endodontics, the final number of schools that were included in the study was twenty.

Respect the use of magnification in pre-clinical and clinical endodontic training (Q1) 

(Table 2), no type of magnification was used in 18 schools (90%), using loupes and operative microscope in 2 of the schools (10%).

**Table 2:**
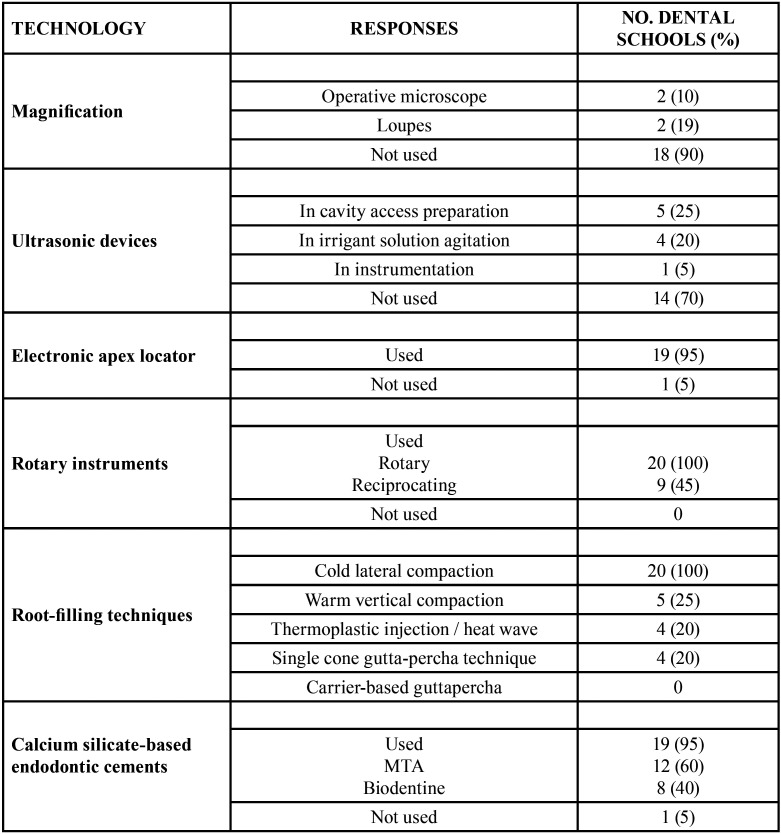
Use of modern technologies in undergraduate Endodontic training in Spain.

The second question (Q2) referred to the use of ultrasound devices during preclinical and clinical practices. In 70% of the schools (n = 14), no type of ultrasonic instrument was used. However, in 5 of the schools (25%) ultrasound was used to prepare the access cavity, and in 4 (20%) to activate the irrigation solution. Only 1 of the schools used ultrasonic in root canal instrumentation.

The use of electronic apex locators in working length determination was the target of the third question (Q3). Nineteen of the schools (95%) used both the radiographic technique and electronic apex locator in working length determination. Only one school exclusively used the radiographic technique.

The fourth question (Q4) was about the use of rotary systems in root canal instrumentation. Rotary instrumentation were used in all schools, being Protaper Next (Dentsply Sirona Endodontics, Ballaigues, Switzerland) and Protaper Gold (Dentsply Tulsa Dental Specialties, Tulsa, OK, USA) the most commonly used rotary systems (45% and 65% of schools, respectively). In nine (45%) of the schools some Reciprocating systems were used in 45% of schools (n = 9), being Reciproc (VDW, Munich, Germany) the most commonly used reciprocating system (5 schools).

The use of filling techniques other than lateral compaction was the objective of the fifth question (Q5). Although cold lateral compaction technique was used in all schools in both preclinical and clinical practices, five schools (25%) also used the warm vertical compaction technique, four schools (20%) used the single cone gutta-percha technique, and four others (20%) also used the thermoplastic injection techniques with continuous heat wave. No school used carrier-based gutta-percha.

The last question (Q6) referred to the use of advanced endodontic materials in pre-clinical and clinical Endodontic training. Calcium silicate-based endodontic cements (CSBEC) were used in 19 of the schools (95%), being MTA (Dentsply, Tulsa Dental, Tulsa, OK, USA) the most used CSBEC (n = 12, 60%), following by Biodentine (Septodont, Saint Maur des Fosses, France), used in eight dental schools (40%).

## Discussion

The objective of this survey was to evaluate the incorporation of contemporary technologies and new materials in endodontics teaching in Spanish dental schools. The results show that Spanish dental schools have only incorporated some of the new endodontic technologies and materials, including the electronic apex locator, rotary instrumentation, and the new bioceramic cements. However, modern root filling techniques, magnification and ultrasonic instruments are not yet used in most dental schools.

The studies on the teaching of endodontics are scarce. In 1997, a survey conducted in the UK, including data gathered by questionnaire from all 14 undergraduate dental schools, analyzed the pattern of undergraduate endodontic teaching the UK (6). At that time, the incorporation of magnification, ultrasound and rotary instruments was still very uncommon, and bioceramic cements were beginning to develop. Posteriorly, other survey analyzed the teaching of endodontics in two European dental schools from Sweden (Malmö University, Malmö), and France (Faculty of Dentistry, Paris 5 University - René Descartes) was analyzed (Petersson *et al.* 2002), but only assessed the quality of treatment in the student clinics. Another study, including data from 27 (96%) of German dental schools, also analyzed the teaching of endodontic clinical practices (Sonntag *et al.* 2008).

The teaching of root canal treatment has been analyzed in several surveys carried out in Europe. A survey evaluated the impact of rotary nickel-titanium (NiTi) instruments on undergraduate teaching in French dental schools (Arbab-Chirani & Vulcain 2004), and another one was conducted in the UK analyzing the requirements of dental undergraduates in the area of root canal treatment (10). Finally, the study of Al Raisi *et al.* (2019), conducted in the United Kingdom, has been the first analyzing all the aspects of endodontic teaching.

Although in recent decades various technologies and new materials have been integrated into the clinical practice of endodontics, few studies have analyzed whether they were also being incorporated into the teaching of endodontics. The results of the present study carried out in Spain provide the first data on the teaching of endodontics within Spain. Moreover, this study, together with the one conducted in the UK (11), show for the first time how new technologies and materials are being incorporated into the undergraduate endodontic teaching.

The questions raised in the present study are based on the survey previously conducted in the UK (11). Taking into account the response rate (91%), similar to that obtained in other surveys conducted in Europe (7–9), the data provided by the present survey are consistent.

Learning endodontic treatment is essential in endodontic training. That is why the largest percentage of time in endodontic studies is dedicated to root canal treatment (11). Determining the working length is a very important phase of root canal treatment, and the electronic apex locator has been widely introduced into everyday clinical practice. The use of electronic apex locators in working length determination is the gold standard technique. The results of the present survey show that, as in the UK (11), electronic apex locators are used in working length determination in all Spanish dental schools.

However, other modern technologies do not appear to have yet been fully incorporated into endodontic teaching. So it is with magnification. Survey results show that only 10% of dental schools use magnification in the practical teaching of endodontics. By contrast, in the UK 80% of dental schools have incorporated the use of magnification (11). A possible explanation for the delay in incorporating magnification into endodontic teaching could be the high economic cost of operating microscopes and the lack of staff training (12).

Another of the new technologies that seems not to have been incorporated into the teaching of endodontics, at the undergraduate level, is ultrasonic devices. Currently, the use of ultrasound is widely introduced in the endodontic clinic for the preparation of the access cavity and the agitation of the irrigating solution during root canal instrumentation. Nevertheless, almost three quarters (70%) of Spanish dental schools do not use ultrasonic instruments in the teaching of endodontics. Only 25% of schools use ultrasonic instruments in endodontic training to prepare the access cavity, and 20% in irrigant solution agitation. In contrast, data from the survey conducted in the UK show that ultrasonic instruments are employed in 53% of schools for access cavity preparation, and in 80% of schools for different procedures during clinical practices (11). The use of ultrasonic instruments is a pending subject in the undergraduate teaching of endodontics in Spain

Although hand instruments continue to be used in all dental schools, all Spanish dental schools taught their students to use rotary instruments during endodontic training. Moreover, reciprocating systems (Reciproc, Wave One) are being used in almost half (45%) of the Spanish dental schools. These data show that dental schools have incorporated rotary instruments in root canal treatment training. Similar results have been obtained in several surveys conducted in other European countries. Fourteen years ago, in France, 81% of undergraduate dental schools used rotary nickel-titanium instruments on undergraduate endodontic teaching (9). Nowadays, in the UK all dental schools used rotary instruments (Al Raisi *et al.* 2019) in endodontics training.

Regarding root canal filling, the results show that cold lateral compaction is, still today, the root-filling technique taught in all Spanish dental schools. On the contrary, cold lateral compaction was used exclusively in 47% of the dental schools in the UK (11). Few dental schools use the warm vertical compaction technique (25%), the thermoplastic injection technique (25%) or the single cone gutta-percha technique (20%). These three techniques are used in the UK by 33%, 13%, and 27% of the schools, respectively (11). It is striking that despite the enormous commercial support for carrier-based gutta-percha systems, none of the Spanish dental schools use it. In contrast, in the UK 27% of schools used carrier-based gutta-percha systems in endodontic teaching (11).

The responses to the last question in the survey demonstrate that the use of calcium silicate endodontic cements is fully incorporated into undergraduate endodontic clinical training in Spain. Majority of English dental schools also are teaching and recommending the application of calcium silicate cements (11).

Of the six new technologies and materials analyzed in this survey, modern root filling techniques, magnification and ultrasonic instruments are not being adequately taught in Spanish dental schools. Undoubtedly, one of the reasons may be the high cost that these three new technologies and materials entail. In fact, most of the dental schools that did include these technologies in undergraduate endodontic teaching were private universities.

Another reason that could explain the little incorporation of some modern technologies to the teaching of endodontics in Spanish dental schools, could be the training of the teachers who teach endodontics, as well as the motivation and interest they have (13). The teaching of endodontics should be supervised by instructors with specialized training in endodontics (1). In the first survey conducted in the UK in 1997, none of the schools had supervising staff with advanced training in endodontics (6). However, in the recent survey carried out in the UK (11), most of dental schools employed general dental practitioners with special interest in endodontics, endodontists or a combination of both for endodontics teaching. The specialty of endodontics is not recognized in Spain, so the professors who teach in dental schools are general dental practitioners with a special interest in endodontics.

## Conclusions

Spanish dental schools have incorporated some of the new endodontic technologies and materials, including the electronic apex locator, rotary instruments, and the new bioceramic cements; however, the modern root filling techniques, magnification, and ultrasonic instruments are not yet used in most dental schools.
